# Expression of pro- and anti-inflammatory cytokines during anti-proprotein convertase subtilisin/kexin type 9 therapy in patients with statin-resistant familial hypercholesterolemia

**DOI:** 10.3389/fcvm.2024.1417044

**Published:** 2024-07-18

**Authors:** Julieta Danira Morales-Portano, Rafael Trujillo-Cortés, Bricia Margarita Roa-Martínez, Rebeca Pérez-Cabeza de Vaca, Silvia García, Paul Mondragón-Terán, Juan A. Suárez-Cuenca

**Affiliations:** ^1^ Cardiology Department, National Medical Center “20 de Noviembre,” ISSSTE, Mexico City, Mexico; ^2^ Coordination of Research, National Medical Center “20 de Noviembre,” ISSSTE, Mexico City, Mexico; ^3^ Clinical Research Department, National Medical Center “20 de Noviembre,” ISSSTE, Mexico City, Mexico

**Keywords:** anti-PCSK9, atherosclerosis, dyslipidemia, hypercholesterolemia, inflammatory cytokines, LDL cholesterol, statin resistance

## Abstract

**Background:**

Some clinical dyslipidemia cases do not respond to statins, known as statin-resistant familial hypercholesterolemia (SR-FH), in which patients are under a high cardiovascular risk despite statin therapy. Therefore, novel therapeutic alternatives are required. Proprotein convertase subtilisin/kexin type 9 inhibitors (PCSK9i) reduce cholesterol levels and cardiovascular disease risk, particularly in patients with SR-FH, where PCSK9i may differentially affect pro- and anti-inflammatory mediators depending on the clinical setting.

**Aim:**

To evaluate the effect of PCSK9i treatment on pro- and anti-inflammatory cytokines in patients with SR-FH.

**Methods:**

Before–after comparison, quasi-experimental, single-center study in patients with SR-FH. Blood samples were processed to obtain complete blood counts of glycated hemoglobin and serum lipid levels. Flow cytometry was performed to characterize baseline circulating M1- and M2-macrophages and monocytes. Multiplexing of plasma samples was used to compare plasma fraktaline, interleukins (ILs), monocyte chemoattractant protein-1 (MCP-1), and tumor necrosis factor (TNF)-alpha. The endpoints were lower serum lipid levels and pro-inflammatory mediator modification.

**Results:**

Twenty patients with SR-FH, aged 58 years and most of them males, were included, with a mean body–mass index of 26.4 and showing ischemic heart disease and similar values of baseline M1- and M2-macrophages and monocytes. Six-month iPSCK-9 therapy considerably reduced LDLc, increased anti-inflammatory cytokine (IL-4), and modified pro-inflammatory cytokine (TNF-alpha and MCP-1) levels. No notable effects were observed for the other markers.

**Conclusion:**

PCSK9i therapy exerted subclinical anti-inflammatory and anti-atherogenic effects, indicating potential benefits for clinical outcomes.

## Highlights

• Atherosclerosis is a major cardiovascular disease-associated pathophysiological mechanism.
• Patients with statin-resistant familial hypercholesterolemia present a high cardiovascular risk despite statin therapy.
• PCSK9 inhibitors effectively reduce LDLc levels.
• PCSK9i therapy increases plasma IL-4 and reduces MCP-1 in patients with SR-FH.
• PCSK9i effect over pro- and anti-inflammatory cytokines suggests potential benefits for atheroma plaque and clinical outcomes.

## Introduction

Cardiovascular diseases are one of the main causes of mortality worldwide, and atherosclerosis is a major associated pathophysiological mechanism. The economic impact of cardiovascular diseases is estimated at approximately 555 billion dollars in the USA, and by 2030, cardiovascular diseases are expected to result in the death of >23.3 million people annually (1, 2).

Atherosclerosis is a chronic disease that is characterized by the deposition of lipids on the arterial wall, along with the activation of the innate and adaptive immune systems. Atherosclerotic plaque formation involves a combination of endothelial dysfunction, extensive intimal lipid deposition, inflammatory response, vascular smooth muscle cell proliferation, and extracellular matrix remodelling (2). During early atherogenesis, Low Density Lipoprotein Cholesterol (LDLc) enhances leukocyte recruitment to the injured areas, wherein endothelial cells increase the secretion of chemokines interleukin (IL)-8, monocyte chemoattractant protein-1 (MCP-1), and growth-regulated protein *α* in response to LDL*α* (3). Such events stimulate the recruitment of T cells, neutrophils, and monocytes. In turn, activated leukocytes produce more chemokines, further amplifying the inflammatory response through IL-6 production in monocytes and lymphocytes, and the promotion of antibody production and induction of C-reactive protein (4).

Reportedly, statins can reduce LDLc levels, stabilize atheromatous plaques, reverse endothelial dysfunction, and decrease thrombogenesis. In contrast, novel pharmacological approaches, such as proprotein convertase subtilisin/kexin type 9 (PCSK9) inhibitors (PCSK9i), have been developed to effectively reduce LDLc levels, showing favourable tolerability and safety profiles (5).

PCSK9, a proprotein convertase 2, is a natural inhibitor of LDL receptor (LDLR) that binds to the extracellular domain of LDLR and triggers its intracellular degradation. PCSK9 and LDLR are coordinately regulated at the transcriptional level by sterols through their sterol regulatory element-binding protein-2 (SREBP-2), embedded in the promoter and are co-induced by statins. PCSK9 is predominantly expressed in adult liver hepatocytes and small intestinal enterocytes. It is synthesized as a 72k zymogen via autocatalytic cleavage in the endoplasmic reticulum. After secretion from the cells, PCSK9 binds to the extracellular epidermal growth factor-A domain of LDLR and triggers its intracellular degradation in lysosomes, thereby increasing LDLc level (6, 7).

Additionally, atherosclerosis is associated with dyslipidemia, which can be modified through lifestyle interventions, and the pharmacological approach is a key therapeutic modality for reducing serum lipids.

In the clinical setting, some dyslipidemia cases show a lack of response to statins, also known as statin-resistant (SR) familial hypercholesterolemia (FH), in which patients maintain a high cardiovascular risk despite statin therapy. Hence, the development of novel therapeutic alternatives is necessary. Reportedly, PCSK9i can reduce cholesterol levels and the risk of cardiovascular diseases, particularly in patients with SR-FH. PCSK9i, as anti-PCSK9 monoclonal antibodies, namely alirocumab and evolocumab, can reduce LDLc by 50%–70%, lowering the probability of atherosclerotic plaque-induced cardioischemic episode development.

Furthermore, PCSK9i may also affect inflammatory mechanisms. Inflammation-regulatory effects of evolocumab were tested in a pilot trial in Chinese population at early stage after acute coronary syndrome. Evolocumab induced a rapid lipid reduction, accompanied by the decrease in pro- and anti-inflammatory circulating cytokines (8). Other inflammation-regulatory effects of PCSK9i have been described in population with FH, such as enhanced activation of T-regulatory cells, increased anti-inflammatory mediators like IL-10, reduction of pro-inflammatory cytokines and impairment in leukocyte attachment to endothelium (9). Since diversity of inflammation-regulatory effects of PCSK9i, which may further depend on the clinical scenario, we evaluated the effects of PCSK9i therapy on pro- and anti-inflammatory circulating cytokines, as well as other inflammation-related markers, in patients with SR-FH.

## Methods

### Study design

Non-randomized, non-controlled, before–after comparison, quasi-experimental, single-center study.

### Study population and treatment

Herein, patients older than 18 years, who were diagnosed with SR-FH and attended at the Cardiology Department, Centro Médico Nacional “20 de Noviembre ISSSTE,” Mexico City were enrolled. FH was defined according to Dutch Score, including cases with total point scores higher than 8, or point scores 6–8, considered as “definite” or “probable” FH, respectively (Dutch Lipid Clinic Network diagnostic criteria for Familial Hypercholesterolemia) (10–12). SR-FH was defined as symptomatic cardiovascular disease accompanied by LDLc concentration of >160 mg/dl, despite a maximally tolerated statin dose (13) in a patient previously diagnosed with FH. Patients with infections, neoplasia, or those undergoing oncological therapy were excluded. Evolocumab (140 mg) was administered intramuscularly every 2 weeks at the discretion of the physician and according to the LDLc response. The protocol was approved by the local Committee for Research, Biosafety and Ethics from Centro Médico Nacional “20 de Noviembre ISSSTE,”; and all patients signed written informed consent for participation as well as the use of blood samples.

### Clinical-Demographic characteristics

Clinical demographic characteristics such as age, sex, past diseases, and medication use were collected through direct interviews and verified through medical records. The body–mass index (BMI) was calculated as weight/height^2^ (kg/m^2^). Blood pressure was measured while the patient was in a seated position, and the mean of three readings taken at 5-min intervals using a Welch Allyn 767 mobile aneroid sphygmomanometer (Welch Allyn Inc., Skaneateles Falls, NY, USA) was considered. Diabetes mellitus was defined according to the guidelines of the American Diabetes Association with one of the following conditions (repeated for confirmation at a separate date): (1) hemoglobin A1C ≥ 6.5%; (2) fasting glucose ≥ 126 mg/dl; or (3) 2-h plasma glucose ≥ 200 mg/dl during an oral glucose tolerance test.

### Complete blood count and lipid profile

Following a 12-h fast, venous blood samples (4 ml) were collected using BD Vacutainer® tubes (Becton, Dickinson & Co., Franklin Lakes, NJ, USA) containing 1.8 mg of ethylenediaminetetra-acetic acid/ml of blood. A standard blood cell count was performed immediately after sample collection. For clinical chemistry, the blood sample was centrifuged at 1,200 g for 5 min at 4°C and separated into fractions. Following this, the plasma was stored at −80°C before analyses. Total blood cell count and plasma triglycerides, total cholesterol, high-density lipoprotein cholesterol (HDLc), and LDLc levels were determined using routine clinical laboratory equipment and a standard auto-analyzer (Synchron CX®9 PRO Clinical System; Beckman Coulter, Brea, CA, USA). The atherogenic index was calculated as log triglyceride/HDLc.

### Flow cytometry

Blood samples were centrifuged using lymphoprep to obtain the leukocyte populations. After three washes, cells were fixed in 4% paraformaldehyde and stored for 12 h. Flow cytometry (MACSQuant Analyzer 10, Miltenyi Biotec) was performed for 1,000,000 events per analysis, and the corresponding isotype controls were used to set the appropriate regions. Cell subpopulations were identified by specific marker combinations (cluster of differentiation [CD]14 + and CD163 + for M1-macrophages; CD14 + and CD206 + for M2-macrophages; and CD16 + and C-X-C motif chemokine receptor [CXCR]4, CD16+, and CXCR2 for monocytes).

### Plasma soluble biomarkers

An aliquot of blood samples was immediately centrifuged at 1,200 g for 5 min and the plasma was collected. Analytes [namely fraktaline, IL-1, IL-4, IL-6, IL-8, IL-10, MCP-1, and tumor necrosis factor (TNF)-alpha] were analyzed using commercially available multiplexing assay (HCYTOMAG-60-08 K, MILLIPLEX MAP Human Cytokine/Chemokine Magnetic Bead Panel—Immunology Multiplex Assay Merc, Millipore, MA, USA), following the instructions of the manufacturer, and the results were read using a Multiplex reader (MAGPIX System, Millipore, Austin, TX, USA).

### Follow-up and study endpoints

Follow-ups were conducted after 6 months of therapy through programmed medical evaluations and blood analyses. The primary endpoint consisted of the modification of serum lipids, and the secondary endpoints included modification in (1) pro-inflammatory mediators [namely neutrophils, lymphocytes, neutrophil-to-lymphocyte ratio (NtLR), and soluble pro-inflammatory cytokines] and (2) atherogenic index.

### Statistical analyses

Quantitative data are presented as the mean ± standard deviation and the categorical data as n(%). A one-way *T*-test was performed. Statistical significance was set at *p* < 0.05.

## Results

**Figure 1:**
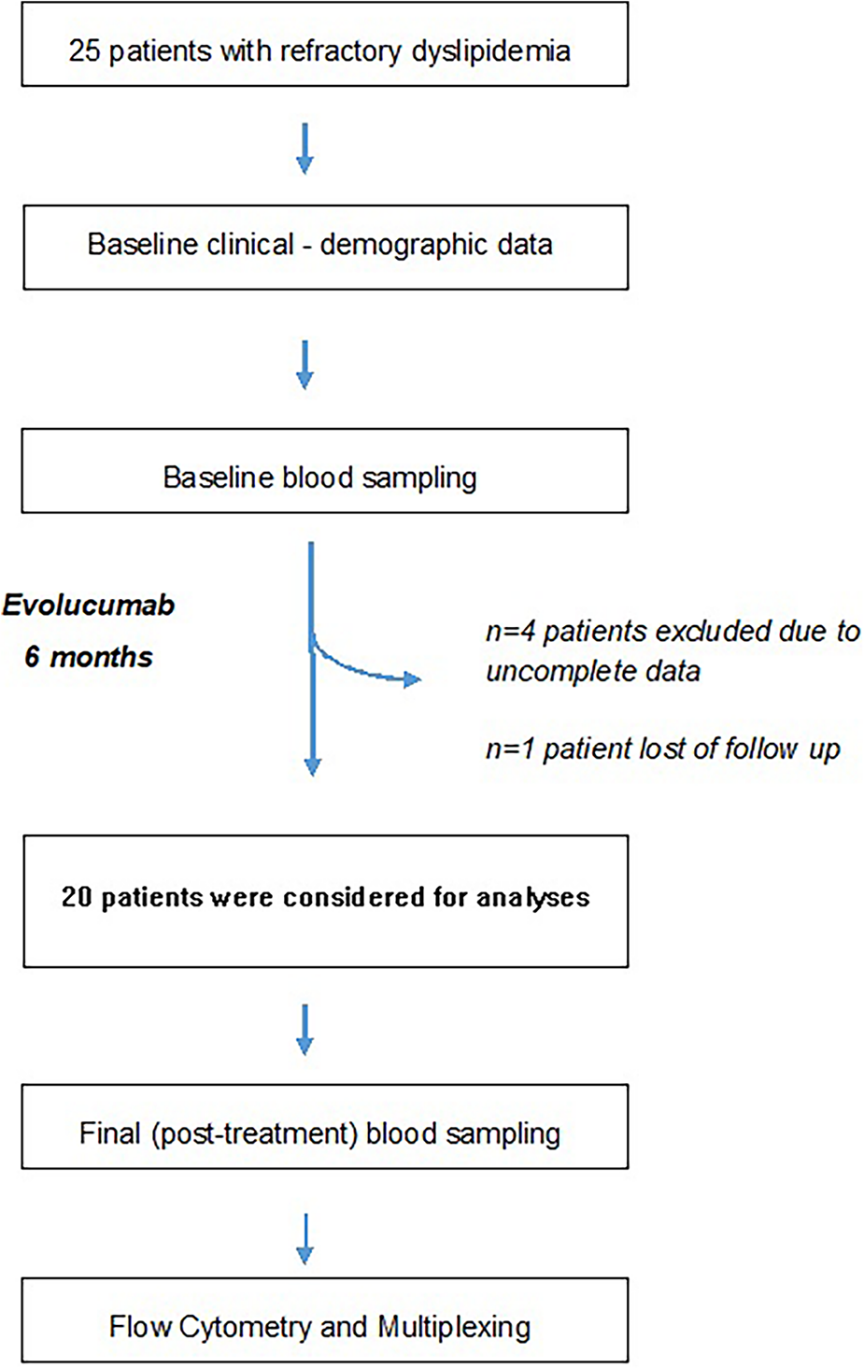
Study flowchart. Refractory dyslipidemia has been defined in Methods.

After the exclusion of five patients (Figure 1, flow chart), a total of 20 patients with refractory dyslipidemia, mainly based on higher LDLc levels, were included in the study; they were aged 58 years, most of whom were males, with a mean BMI of 26.4, and with ischemic heart disease. Furthermore, the patients presented borderline levels of subclinical inflammatory markers and a high cardiovascular risk (Table 1).

**Table 1:** Clinical-demographic characteristics of the study population (*n* = 20).

Age (years old)	58.5	±15.1
Male Sex	12	60%
Weight (kg)	70.3	±14.9
Height (m)	1.62	±0.08
BMI (kg/m^2^)	26.4	±4.91
Co-morbidities		
Nonischemic^a^	6	30%
Ischemic *dyslipidemia*	2	10%
*dyslipidemia and/or Diabetes and/or Hypertension*	10	50%
*Dyslipidemia and others*^b^	2	10%
Neutrophils	1.95	±0.56
Lymphocytes	2.02	±0.69
NtLR	0.61	±0.05
% CD14/CD163 Macrophages (M1)	3.81	±1.20
% CD14/CD206 Macrophages (M2)	40.1	±6.8
% CD16/CXCR4 Receptor CXCR4 (Monocytes)	38.7	±5.5
% CD16/CXCR2 Receptor CXCR2 (Monocytes)	45.8	±5.2
Total Cholesterol (mg/dl)	260.2	±211.6
HDLc (mg/dl)	51.4	±39.1
LDLc (mg/dl)	130.1	±55.6
Triglycerides (mg/dl)	192.3	±108.6
Atherogenic index	0.61	±0.05

NtLR, neutrophil-to-lymphocyte ratio; CD, cluster of differentiation; CXCR, C-X-C motif chemokine receptor; HDLc, high-density lipoprotein cholesterol; LDLc, low-density lipoprotein cholesterol.

^a^ Denotes myocardiopathy, obesity, diabetes mellitus, systemic arterial hypertension, chronic liver disease, and hyperuricemia.

^b^ Denotes depression, hypothyroidism, rheumatoid arthritis, and asthma.

**Figure 2:**
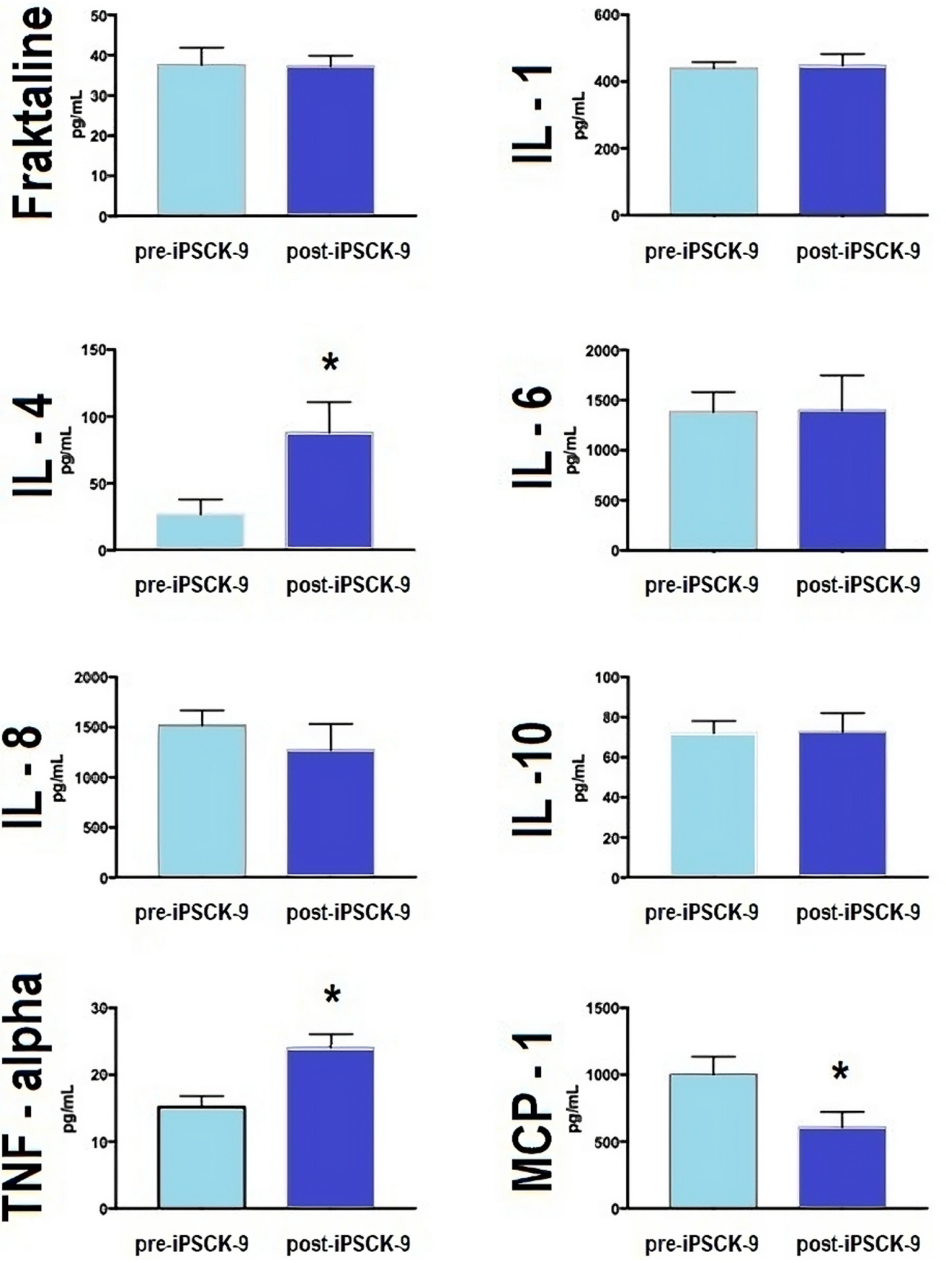
iPSCK9 therapy and pro- and anti-inflammatory plasma cytokines. Bar graphs comparing plasma concentration of pro-inflammatory (IL-1, IL-6, TNF, and MCP-1) and anti-inflammatory (IL-4 and IL10) cytokines, according to time phases: pre-iPSCK9 or after 6 months of iPSCK9 therapy. The mean comparison was made by a one-way, independent *T*-test. Fraktaline (*p*-value > 0.05), IL-1 (*p*-value > 0.05), IL-4 (*p*-value = 0.03), IL-6 (*p*-value > 0.05), IL-8 (*p*-value > 0.05), IL-10 (*p*-value > 0.05), TNF-alpha (*p*-value = 0.001), and MCP-1 (*p*-value = 0.03). IL, interleukin; TNF, tumor necrosis factor; MCP-1, monocyte chemoattractant protein-1; and iPSCK-9, iPSCK9, proprotein convertase subtilisin/kexin type 9 inhibitor.

Six-month iPSCK9 therapy considerably reduced LDLc (Table 2); which was accompanied by a significant increase of about 3 fold in anti-inflammatory cytokine IL-4; as well as a significant modification of pro-inflammatory cytokines MCP-1 (about 50% decrease) and TNF-alpha (about 50% increase) levels. No significant effects were observed for other markers (Figure 2).

**Table 2:** Comparison of pre- vs. post-proprotein convertase subtilisin/kexin type 9 inhibitor therapy.

	Pre-PSCK9	6 m post-iPSCK9	*p*-value
Cholesterol	260.2 ± 211.6	216.7 ± 157.6	0.26
HDLc	51.4 ± 39.1	51.7 ± 31.4	0.58
LDLc	130.1 ± 55.6	96.9 ± 55.9	0.05
Triglycerides	192.3 ± 108.6	161.5 ± 68.4	0.18
Neutrophils	3.81 ± 1.20	4.41 ± 2.15	0.18
Lymphocytes	1.95 ± 0.56	1.89 ± 0.61	0.37
NtLR	2.06 ± 0.69	2.44 ± 1.18	0.16
Atherogenic Index	0.61 ± 0.05	0.59 ± 0.05	0.43

The mean comparison was made by a one-way, independent *T*-test.

6 m, 6 months; HDLc, high-density lipoprotein cholesterol, LDLc, low-density lipoprotein cholesterol; NtLR, neutrophil-to-lymphocyte ratio; and iPSCK9, proprotein convertase subtilisin/kexin type 9 inhibitor.

## Discussion

Herein, the results showed that iPSCK9 therapy considerably decreased LDLc levels in patients with SR-FH. Additionally, iPSCK9 therapy affected the expression of plasma cytokines with pro- and anti-inflammatory effects, potentially influencing the atherogenic process in a population without current acute coronary syndrome.

The LDLc-reducing effect of iPSCK9 observed in this study is consistent with the findings of other studies, systematic reviews, and meta-analyses (14). Herein, a 31% LDLc reduction was achieved after 6 months of follow-up. Similarly, other studies have achieved notable LDLc reductions as early as 12 weeks of therapy, and some studies have reported a 60% LDLc reduction at 4 weeks of therapy in a similar Latin-American population (15). Furthermore, iPSCK9 have also been described to stabilize atherosclerotic plaque in patients with cardiovascular disease. Although precise mechanism has not been elucidated, evolucumab has elicited target LDL-C levels as well as reduction of plaque neovascularization, as assessed by coronary diagnosis imaging. This becomes relevant since neovascularization is one of the characteristics of vulnerable plaque, whereas PSCK9i and statin therapy have shown different neovascularization-modulating effects (16–19).

Likewise, the switch of macrophage characteristics from pro-inflammatory to anti-inflammatory phenotype and the activation of inflammation-resolving mechanisms may have a key role in PSCK9i plaque-modulating effects (20). Therefore, we determined baseline macrophage profile and inflammatory markers in our study population, where data suggest a low level of inflammation in the study population, as indicated by the proportion of macrophages M1:M2 and NtLR. Consistently, disrupted pro-inflammatory markers have been reported in populations with FH (21), which may be considered a subclinical cardiovascular risk.

Along with LDLc reduction, PSCK9i therapy induced higher plasma concentrations of the anti-inflammatory cytokine IL-4 and reduced that of the pro-inflammatory and pro-atherogenic cytokine MCP-1, suggesting an overall inflammation-limiting effect. Anti-inflammatory effects have been previously described in evolocumab-treated patients (22, 23), which are probably mediated by sirtuin3 (24). This finding is relevant because iPSCK9 therapy may present several beneficial effects other than LDLc reduction, including inflammatory-modulation effects and subclinical anti-atherogenic properties.

However, iPSCK9 therapy may result in different effects regarding pro-and anti-inflammatory mechanisms, which may be explained by the diversity of clinical scenarios and/or study designs. For example, Evolocumab similary decreased pro- and anti-inflammatory circulating cytokines in a pilot study of Chinese population after acute coronary syndrome (8); while PCSK9i therapy has shown to increase anti-inflammatory mediators like IL-10; to reduce of pro-inflammatory cytokines and to impair the ability of leukocytes to attach endothelium, as well as promoting activation of T-regulatory cells in population with FH (9). In the present study, IL-4 was the only anti-inflammatory cytokine that significantly increased in response to PCSK9i, suggesting a potential role regarding anti-inflammatory effect of IPCSK9 therapy. Comparatively, IL-6 and IL-10 did not show significant modification; while other anti-inflammatory cytokines like IL-11 and IL-13 were missing due to the characteristics of the expression panel arrange used.

Likewise, iPCSK9 therapy resulted in elevated plasma TNF-α, which reflects the complex specific relationship between these molecules, leading to variable responses depending on specific contexts and underlying conditions. Although, iPCSK9 is generally associated with anti-inflammatory effects, there are reports that iPCSK9 increases TNF-α levels, which may be explained by either compensatory mechanisms, where the body initiate adaptive inflammatory responses facing PCSK9 inhibition; or different cell types might respond differently to PCSK9 inhibition; both mechanisms potentially leading to transient increase in TNF-α (25–29). Moreover, factors like the study design, the specific conditions of the patients and the methods used to measure pro-inflammatory mediators may influence final outcome.

Interestingly, PCSK9i has been described to lower TLR expression, as well as pro-inflammatory mediators IL-17, IL-1β, ICAM and VCAM, which parallels the reduction of arterial intimal thickness and atherogenic plaques; with a higher effect as compared to statins, in animal model. While such anti-inflammatoty effects may be potientiated by the combination of PCSK9i—statins (30).

Finally, some limitations of the present should be considered, such as: (1) the small sample size and related bias; (2) the lack of multivariate analyses; (3) methodological restrictions, including the limited number of pro- and anti-inflammatory cytokines evaluated, due to the panel arrange used, as well as (4) the missing effect of IPCSK9 on the proportion macrophages M1:M2, which would have been highly revealing, but not able to determine due to planning inconvenients.

Future perspectives for PCSK9i therapy involve deeper insights into several benefits beyond LDL-C reduction, such as elucidating the mechanisms underlying pro- and anti-inflammatory effects, as well as its role on atherogenesis and plaque stabilization. Likewise, exploration of potential synergistic effects with other anti-inflammatory agents leading to more effective modulation of inflammation, potentially broadening therapeutic indications to non-cardiovascular, metabolic diseases such as Metabolic Syndrome and Diabetes**,** throughout specifically-designed clinical trials that could provide robust data to support these perspectives.

To the best of our knowledge, this is one of the first studies characterizing baseline inflammatory profile in patients with SR-FH, as well as to report balance of pro- and anti-inflammatory cytokines because of PSCK9i therapy, which may further impact on the atherogenic, plaque and cardiovascular risk, particularly relevant in population with SR-FH; which need to be explored further.

## Conclusions

Altogether, iPCSK9 therapy notably reduces LDLc and plasma IL-4 and MCP-1 levels, exhibiting subclinical anti-inflammatory and atherogenic effects, and indicating potential benefits for clinical outcomes in population with SR-FH.

## Data Availability

The original contributions presented in the study are included in the article/Supplementary Material, further inquiries can be directed to the corresponding author.
